# Examining the clinical and genetic spectrum of maturity-onset diabetes of the young (MODY) in Iran

**DOI:** 10.1038/s41598-024-70864-y

**Published:** 2024-08-27

**Authors:** Sara Asgarian, Hossein Lanjanian, Shiva Rahimipour Anaraki, Farzad Hadaegh, Maryam Moazzam-Jazi, Leila Najd-Hassan-Bonab, Sajedeh Masjoudi, Asiyeh Sadat Zahedi, Maryam Zarkesh, Bita Shalbafan, Mahdi Akbarzadeh, Sahand Tehrani Fateh, Davood Khalili, Amirabbas Momenan, Narges Sarbazi, Mehdi Hedayati, Fereidoun Azizi, Maryam S. Daneshpour

**Affiliations:** 1 Cellular and Molecular Endocrine Research Center, Research Institute for Endocrine Sciences, Shahid Beheshti University of Medical Sciences, P.O. Box 19195-4763, Tehran, Iran; 2 Faculty of Medicine, Iran University of Medical Sciences (IUMS), Tehran, Iran; 3 Prevention of Metabolic Disorders Research Center, Research Institute for Endocrine Sciences, Shahid Beheshti University of Medical Sciences, Tehran, Iran; 4 Clinical Research Development Center of Labbafinejad Hospital, Shahid Beheshti University of Medical Sciences, Tehran, Iran; 5 School of Medicine, Tehran University of Medical Sciences, Tehran, Iran; 6 Endocrine Research Center, Research Institute for Endocrine Sciences, Shahid Beheshti University of Medical Sciences, P.O. Box 19195-4763, Tehran, Iran

**Keywords:** Maturity-onset diabetes of the young, MODY, Monogenic diabetes, *HNF1A*, *HNF4A*, *GCK*, DNA, Genetics, Genetic markers, Genotype, Endocrinology, Diagnosis, Genetic testing

## Abstract

Maturity-onset diabetes of the young (MODY) is an uncommon monogenic type of diabetes mellitus. Detecting genetic variants for MODY is a necessity for precise diagnosis and treatment. The majority of MODY genetic predisposition has been documented in European populations and a lack of information is present in Iranians which leads to misdiagnosis as a consequence of defects in unknown variants. In this study, using genetic variant information of 20,002 participants from the family-based TCGS (Tehran Cardiometabolic Genetic Study) cohort, we evaluated the genetic spectrum of MODY in Iran. We concentrated on previously discovered MODY-causing genes. Genetic variants were evaluated for their pathogenicity. We discovered 6 variants that were previously reported in the ClinVar as pathogenic/likely pathogenic (P/LP) for MODY in 45 participants from 24 families (*INS in 21 cases, GCK in 13, HNF1B in 8, HNF4A, HNF1A, and CEL in 1 case).* One potential MODY variant with Uncertain Risk Allele in ClinVar classification was also identified, which showed complete disease penetrance (100%) in four subjects from one family*.* This is the first family-based study to define the genetic spectrum and estimate the prevalence of MODY in Iran. The discovered variants need to be investigated by additional studies.

## Introduction

Maturity-onset diabetes of the young (MODY) is an uncommon monogenic type of diabetes mellitus that occurs in around 1–5% of diabetes cases globally^1^. MODY is brought on by dominantly acting, heterozygous variants in genes required for pancreatic β-cell function^2^. Early detection of MODY through accurate genetic testing leads to better long-term management, genetic counseling, and family screening, with reduced complications and psychological distress^3–5^. To this point, numerous distinctive subtypes of MODY have been identified based on the primary genetic mutation, with varying genomic heterogeneity, hyperglycemia patterns, and treatment responses^3,6–8^. MODY accounts for the majority of monogenic diabetes traits, with the classic characteristic of young-onset diabetes, and lacks both type 1 and type 2 diabetes (T1DM, T2DM) hallmarks along with a family history of diabetes in a parent and first-degree relatives of that afflicted parent^3,9^. Contrary to T1DM and T2DM, however, molecular genetic testing is sensitive and specific for MODY diagnosis, clinical course prediction, and follow-up^3–5^. Additionally, as MODY is transmitted in an autosome dominant (AD) pattern, it frequently leads to genetic testing in other family members with hyperglycemia who may also be carriers of a variant, helping clarify the diabetes classification^3^. The aforementioned features, along with a general lack of knowledge, hinder clinical diagnosis, resulting in the first misdiagnosis of T1DM or T2DM in the majority of children with monogenic diabetes^3,10^.

According to the second international consensus report on precision diabetes medicine in 2023, there is a critical gap in the literature for the genetic testing of monogenic diabetes mellitus (MDM) to reduce concerns regarding health disparities with replication and external validation in non-European ancestry populations^11^. Despite earlier linkage analysis for MODY, there are still diagnosed genetically unexplained cases^12^. Various studies indicate that there are significant differences in the prevalence of MODY variants among distinct ethnic groups^13^. To date, most research has investigated genetic causes of MODY in people of European descent, with only a few studies undertaken in the Middle East^14–16^. Considering the high rate of consanguineous marriages and the significant load of genetic homozygosity, studies on this region are essential to discovering MODY-associated variants^5,16^.

## Material and methods

### Cohort characterization

Study participants were from the Tehran Cardiometabolic Genetic Study (TCGS), a 25-year-old large-scale family-based longitudinal cohort study. TCGS is a representative of the Iranian population from all Iranian ethnicities, as well as from long-term residents (≥ 25 years), with follow-ups scheduled every 3 years. The ethics committee approved all procedures performed in this study on human subject research at the Research Institute for Endocrine Sciences, Shahid Beheshti University of Medical Sciences (code of “IR.SBMU.ENDOCRINE.REC.1395.366″), which were following the 1964 Helsinki Declaration and its later amendments or comparable ethical standards. Informed consent was obtained from all participants before inclusion in the study at each survey. Subjects were followed up with an extensive medical examination, questionnaires, and a venous blood sample using standard protocols^17^^18^. Fasting Plasma Glucose (FPG) and Oral Glucose Tolerance Test (OGTT) were measured by the enzymatic colorimetric glucose oxidase method (Pars Azmoon Inc., Tehran, Iran)^19,20^. More details for other assessments are available in the cohort descriptive article^20,21^. The Body Mass Index (BMI) Cut-off determined by the World Health Organization (WHO) growth standards^22,23^.

### Phenotype classification

Study participants were identified as diabetes, pre-diabetes, or non-diabetes based on blood biomarkers (FPG, OGTT) or self-reported administration of anti-diabetes medications. Glycemic status was determined according to American Diabetes Association (ADA) standards^24^. A person was classified as having diabetes if they matched one of the following criteria: (1) FPG $\ge$ 126 mg/dl (2) OGTT $\ge$ 200 mg/dl (3) Use of glucose‐lowering agents. For further clarification on glucose‐lowering agents, self-reported questionnaires were used to record medication consumption. Individuals who reported using Metformin for non-diabetic purposes were excluded. Cases with (1) FPG of 100–125 mg/dl or (2) OGTT of 140–199 mg/dl were classified as pre-diabetic. Individuals were classified as non-diabetic when (1) FPG < 100 mg/dl and (2) OGTT < 140 mg/dl. MODY phenotype was suspected if: (1) The diabetes age of onset is under 25 (2) had a family history of diabetes, and (3) absence of evidence to suggest a diagnosis of T1DM or T2DM (normal body weight, absence of acanthosis nigricans, and no evidence of insulin resistance)^3,25^. For our circumstances, the HbA1C test and C-peptide levels were not accessible.

### Genotyping

For DNA preparation, the peripheral blood sample was drawn based on international protocols, and DNA was extracted by Proteinase K, salting out the standard method. The quality and quantity of extracted DNA were evaluated. Samples (n = 16,226) were genotyped by the deCODE genetics company (Iceland) using Illumina Human OmniExpress-24-v1-0 bead chip containing 652,919 single nucleotide polymorphisms (SNPs) loci according to the manufacturer’s specifications (Illumina Inc., San Diego, CA, USA). Among them, 1500 samples were selected for whole genome sequencing with HiSeq X Ten (Illumina) with a minimum average coverage of 30 × . In addition, the un-genotyped variants in the SNP array were filled through imputation using the available whole-genome sequencing data. At every stage, relevant quality control processes were taken into consideration to produce high-quality variants. Details of whole genome sequencing and quality control measures have been described previously and reads were aligned to the GRCh38 reference genome^20,26^. A combined variant call file (gVCF) was generated for all study subjects, which contained all genetic variations detected in the TCGS study participants. The sequence variants were annotated using the last version of Variant Effect Predictor (VEP, ver 105)^20,27^.

### Screening for previously reported MODY-causing variants

The ClinVar database's variants for "Maturity onset diabetes of the young" were the starting points. The following steps were performed for extracting previously reported MODY variants: (1) Genes known to be associated with MODY (*HNF4A, GCK, HNF1A, PDX1, HNF1B, NEUROD1, CEL, INS, ABCC8, KCNJ11, APPL1, RFX6, and NKX6-1*) were selected^3,28^. (2) Variations with clinical significance of Pathogenic/likely pathogenic (P/LP) were included (based on the criteria published by the American College of Medical Genetics and Genomics and the Association for Molecular Pathology (ACMG/AMP), along with ClinGen's terms for low penetrance variants and risk alleles). (3) Available variants in TCGS genetic data were extracted. (4) Genetic variants with minor allele frequency (MAF) < 0.0015% in TCGS data and mean sequencing depth > 20 × were included. The final findings were verified using GnomAD V4.1.0, and the total allele frequencies were reported.

### Potential MODY-causing variants finding

All variants available in TCGS data on the mentioned MODY genes were extracted and filtered for minor allele frequency (MAF) < 0.001% in TCGS and mean sequencing depth > 20 × . Subsequently, potentially MODY-causing variants for the Iranian population were identified based on a Combined Annotation-Dependent Depletion (CADD) Phred score > 20 and complete disease penetrance (100%)^29^. The penetrance of each variant was determined by dividing the number of carriers with diabetic phenotype by the total number of variant carriers (N).

### Familial patterns

All carriers for MODY-causing variants were evaluated for clinical characteristics. Based on the ISPAD guideline 2022, carriers were assessed for MODY phenotype and more detailed clinical features of distinct forms of MODY separately^17,18^. These features included mild/severe hyperglycemia, age of diabetes onset, presence of microvascular complications, dyslipidemia, renal cysts, genital malformations, macrosomia, etc.^3^. Moreover, the pedigree of each MODY candidate case was obtained, and transmission of the variant was evaluated to find the family genetic pattern of the variant and similar clinical features in the other family members^3^.

### Ethical approval and consent to participate

The study was approved by the National Committee for Ethics in Biomedical Research of Iran in December 2012. All procedures were under the ethical standards of the ethics committee on human subject research at the Research Institute for Endocrine Sciences, Shahid Beheshti University of Medical Sciences (code of “IR.SBMU.ENDOCRINE.REC.1395.362”) and with the 1964 Helsinki Declaration and its later amendments or comparable ethical standards.

### Consent for publication

As the corresponding author, I confirm that the manuscript has been read and approved for submission by all the named authors. We declare that this manuscript is original, has not been published before, and is not currently being considered for publication elsewhere.

## Results

### Population characteristics

The characteristics of participants are demonstrated in Table 1. Initially, 20,002 participants from TCGS families were included in the study. The cohort is female-dominated (54.4%), with a mean age of 46.3 ± 20.2 years. Concerning the glycemic status, 3,043 participants (15.2%) were classified as diabetic, 5835 (29.1%) were classified as pre-diabetics, and 11,124 (55.6%) were classified as non-diabetics. Analysis of diabetic cases phenotypically showed that females accounted for 56% of all diabetic participants. The average age of the diabetic participants was 65.4 $\pm$ 14.7 years. Participants with diabetes showed a mean BMI of 29.8 $\pm$ 5.3 kg/${\text{m}}^{2}$, which is higher in comparison with 25.9 $\pm$ 5.0 kg/${\text{m}}^{2}$, for normoglycemic participants.

**Table 1:** Demographic and clinical characteristics of Tehran Cardiometabolic Genetic Study (TCGS) cohort.

	All	Diabetics	Pre-diabetics	Non-diabetics
Number of subjects, n (%)	20,002	3043 (15.2)	5835 (29.1)	11,124 (55.6)
Age, y	46.3 ± 20.2*	65.4 ± 14.7	51.5 ± 19.1	38.4 ± 17.6
Female, n (%)	10,883(54.4)	1707(56.0)	2888 (49.4)	6288 (56.5)
BMI (kg/${\text{m}}^{2}$)	27.4 ± 5.3	29.8 ± 5.3	28.4 ± 5.1	25.9 ± 5.0
Laboratory data (mg/dl)				
FPG	98.4 ± 28.2	137 ± 51.4	95.9 ± 9.3	87.0 ± 6.3
OGTT	118 ± 44.1	211 ± 79.9	125 ± 31.5	98.3 ± 19.2
Triglyceride	134 ± 86.4	166 ± 121	145 ± 82.6	115 ± 68.0
Cholesterol	182 ± 39.9	180 ± 44.5	189 ± 39.9	178 ± 37.4
HDL cholesterol	47.5 ± 11.1	45.2 ± 10.8	46.6 ± 11.0	49.0 ± 11.1
LDL cholesterol	98.6 ± 33.8	93.2 ± 37.6	104 ± 33.9	96.2 ± 31.7
Blood pressure (mmHg)				
SBP	114 ± 18.1	126 ± 19.2	117 ± 17.9	108 ± 14.9
DBP	75.4 ± 10.6	77.9 ± 10.9	77.1 ± 10.4	73.3 ± 10.4
Glycemic status (mg/dl) †				
FPG		147.6 ± 56.5		
OGTT				
DM treatment, n (%)		246.5 ± 83.5		
Insulin		298 (9.70)		
Sulphonylurea		1207 (39.6)		
Metformin		1379 (45.3)		
AGIs		17 (0.50)		
Others		7 (0.00)		

BMI, body mass index. FPG, fasting plasma glucose. OGTT, oral glucose tolerance test. HDL, high-density lipoprotein. LDL, low-density lipoprotein. SBP, systolic blood pressure. DBP, diastolic blood pressure. DM, diabetes mellitus. † The reported laboratory data pertain to each diabetic participant’s onset of diabetes. AGIs, Alpha-glucosidase inhibitors. * Mean ± SD.

### Screening for previously reported MODY-causing variants

Using ClinVar database results (2022-11-17) for "Maturity onset diabetes of the young" to identify candidate variants on MODY genes, 3040 variants were extracted. Among these variants, 688 were available in the TCGS gVCF database. After filtering variants with MAF < 0.0015%, mean sequencing depth > 20 × , and clinical significance of pathogenic/likely pathogenic (P/LP), six variants on six different MODY genes were retained. Table 2 depicts the features of detected variants. Forty-five participants from 24 families carried the aforementioned six variants. Table 3 demonstrates the MODY carrier’s laboratory data, diabetes family history, and more detailed features**.**

**Table 2:** Information of MODY variants in the Tehran Cardiometabolic Genetic Study (TCGS) cohort.

MODY Subtype	chr:position:ref:alt	HGVS nomenclature	rs	Protein change	MAF-TCGS	Allele frequency-GnomAD	Impute info	Penetrance (%)	Diagnosis (n)	CADD phred	Consequence	ClinVar-clinical significance	REVEL	Alpha missense
*HNF4A-MODY*	chr20:44,413,696:G:A	NM_175914.5(HNF4A):c.322G > A	rs377476335	p.Val108Ile	0.000328	0.000009932	0.853567	100	DM	22.2	Missense	P	0.804	P
*GCK-MODY*	chr7:44,149,764:G:C	NM_000162.5(GCK):c.675C > G	rs772754004	p.Ile225Met	0.000711	6.196e-7	0.879049	61.5	DM (3) Pre DM (5) N.D (4) N.A (1)	6.324	Missense	P	0.805	P
*HNF1A-MODY*	chr12:120,994,313::C	NM_000545.8(HNF1A):c.863_864insC	rs766191969	p.Pro289fs	0.000355	0.00009529	0.992043	100	DM	–	Frameshift	P	–	–
*HNF1B-MODY*	chr17:37,739,468:G:A	NM_000458.4(HNF1B):c.516C > T	rs764561297	p.Tyr172 =	0.001422	0.00001487	0.954562	12.5	DM (1) Pre DM (5) N.D (2)	9.416	Synonymous	P	–	–
*HNF1B-MODY*	chr17:37,731,615:G:A **†**	NM_000458.4(HNF1B):c.1025C > T	rs780035561	p.Ser342Phe	0.000355	0.00004091	0.851708	100	DM	20.7	Missense	URA	–	B
*CEL-MODY*	chr9:133,071,270:C:CC	NM_001807.6(CEL):c.1776dup	rs193922638	p.Val593fs	0.000423	0.000004028	0.981079	0	Pre DM	17.15	Frameshift	LP	–	–
*INS-MODY*	chr11:2,160,956:G:A	NM_000207.3(INS):c.16C > T	rs121908278	p.Arg6Cys	0.001422	0.00001365	0.869638	38	DM (8) Pre DM (8) N.D (3) N.A (2)	21.2	Missense	P	0.458	B

^†^Demonstrates the potentially causal MODY variant which showed complete disease penetrance (100%) and all the variant carriers were classified as diabetics based on phenotype data. Chr:Pos, Chromosome:Position of identified variant based on genome build GRCh38. Ref, reference allele. Alt, Alternative allele. MAF, Minor Allele Frequency. Penetrance is shown as the percentage of variant carriers with diabetes. Diagnosis is based on phenotype data. N, Number of subjects. DM, Diabetes Mellitus. Pre DM, Pre-diabetes. N.D, Non-Diabetics. N.A, Not Available glycemic data. CADD, Combined Annotation-Dependent Depletion Phred score. URA, Uncertain risk allele. P, Pathogenic. LP, Likely Pathogenic. B, Benign.

**Table 3:** Clinical characterization and laboratory data of index MODY carriers.

	Previously-reported variants						Potentially-causal variant
MODY subtype	*HNF4A-MODY*	*GCK-MODY*	*HNF1A-MODY*	*HNF1B-MODY*	*CEL-MODY*	*INS-MODY*	*HNF1B-MODY*
Case n/ family n†	1/1	13/7	1/1	8/3	1/1	21/11	4/1
Age, y	33	48.0 ± 21.8*	24	47.8 ± 15.7	43	49.7 ± 15.8	58.7 ± 17.7
Female, n (%)	1 (100)	5 (38.4)	1 (100)	6 (75)	1 (100)	12 (57.1)	2 (50)
BMI (kg/${\text{m}}^{2}$)	22.2	26.5 ± 3.3	24.7	25.8 ± 4.9	32.2	27.6 ± 4.8	30.1 ± 4.4
Underweight %	–	–	–	–	–	–	–
Normal %	100	33.3	100	40	–	46.6	–
Overweight %	–	44.4	–	40	–	33.3	50
Obese %	–	22.2	–	20	100	20.0	50
Glycemic status, n (%)							
Diabetic	1 (100)	2 (16.6)	1 (100)	1 (12.5)	–	3 (15.7)	4 (100)
Pre-diabetic	–	6 (50.0)	–	5 (62.5)	1 (100)	8 (42.1)	–
Non-diabetic	–	4 (33.3)	–	2 (25.0)	–	8 (42.1)	–
Missing	–	1	–	–	–	2	–
Laboratory Data							
FPG (mg/dl)	130	120 ± 65.3	171	95 ± 13.7	101	103 ± 44.0	152 ± 41.3
OGTT (mg/dl)	256	174 ± 135	260	155 ± 83.3	138	128 ± 38.5	231 ± 138
Triglyceride (mmol/l)	109	156 ± 100	158	95.5 ± 43.1	200	158 ± 82.5	179 ± 34.1
Cholesterol (mmol/l)	196	186 ± 50.2	150	196 ± 61.8	239	194 ± 38.6	180 ± 52.5
HDL cholesterol (mmol/l)	43	42.4 ± 8.8	46	55.3 ± 5.6	54	46.2 ± 10.9	33 ± 8.2
LDL cholesterol (mmol/l)	124	103 ± 40	60	111 ± 52.5	142	107 ± 35.4	101 ± 48.3
Serum creatinine (μmmol/l)	1.1	1 ± 0.1	0.8	0.9 ± 0.09	0.8	1.13 ± 0.1	1.25 ± 0.5
Blood pressure (mmHg)							
SBP	68	74.2 ± 6	75	74.7 ± 7.4	89	72.9 ± 9.8	90.5 ± 10.1
DBP	94	118 ± 22.5	100	122 ± 19.5	131	111 ± 17.1	154 ± 13.1
Family history of diabetes ‡							
First-degree relative	1 (100)	–	1 (100)	–	–	2 (66.6)	4 (100)
Second-degree relative	1 (100)	–	1 (100)	–	–	–	4 (100)
DM onset glycemic status ‡§							
FPG (mg/dl)	101	241 ± 89.0	128	130	–	182 ± 104	169 ± 38.2
OGTT (mg/dl)	228	401 ± 174	232	256	–	217 ± 8.7	249 ± 42.4
Treatment (n and %) ‡							
Insulin	–	–	–	–	–	–	1 (25)
Sulphonylurea	–	–	–	–	–	1 (33.3)	3 (75)
Metformin	–	1 (50)	–	1 (100)	–	–	2 (50)

^†^Case n/Family n: number of MODY carriers/number of carrier’s families. The reports for MODY *HNF4A, HNF1A* and *CEL* subtypes don’t contain mean and standard deviation due to only one proband. N, Number of subjects. BMI, Body Mass Index. FPG, Fasting Plasma Glucose. OGTT, Oral Glucose Tolerance Test. HDL, High-Density Lipoprotein. LDL, Low-Density Lipoprotein. SBP, Systolic Blood Pressure. DBP, Diastolic Blood Pressure. DM, Diabetes Mellitus. ‡ The reported data are only among the carriers with diabetic phenotype. § The reported laboratory data pertain to each diabetic participant’s onset of diabetes. * Mean ± SD.

### Potential MODY-causing variants finding

We filtered out all the available variants on mentioned MODY genes in TCGS data which brings 526,569 variants. Based on MAF < 0.001%, mean sequencing depth > 20 × , and a CADD Phred score > 20, 98 variants remained. After evaluating the disease penetrance, complete disease penetrance (100%) was observed in one variant in *HNF1B-MODY* (Table 2). This variant is classified with Uncertain Risk Allele for MODY in ClinVar database. This missense MODY variant was detected in four individuals from one family (Table 3). Notably, all carriers were diabetic based on their phenotype data.

### Familial pattern

Regarding the clinical characteristics and disease transmission in MODY carriers’ pedigree, the result for each subtype of MODY is mentioned below. For some subtypes of the disease, we could not find any variant in our population. The rest will be explained in detail:

#### HNF4A-MODY

**Figure 1:**
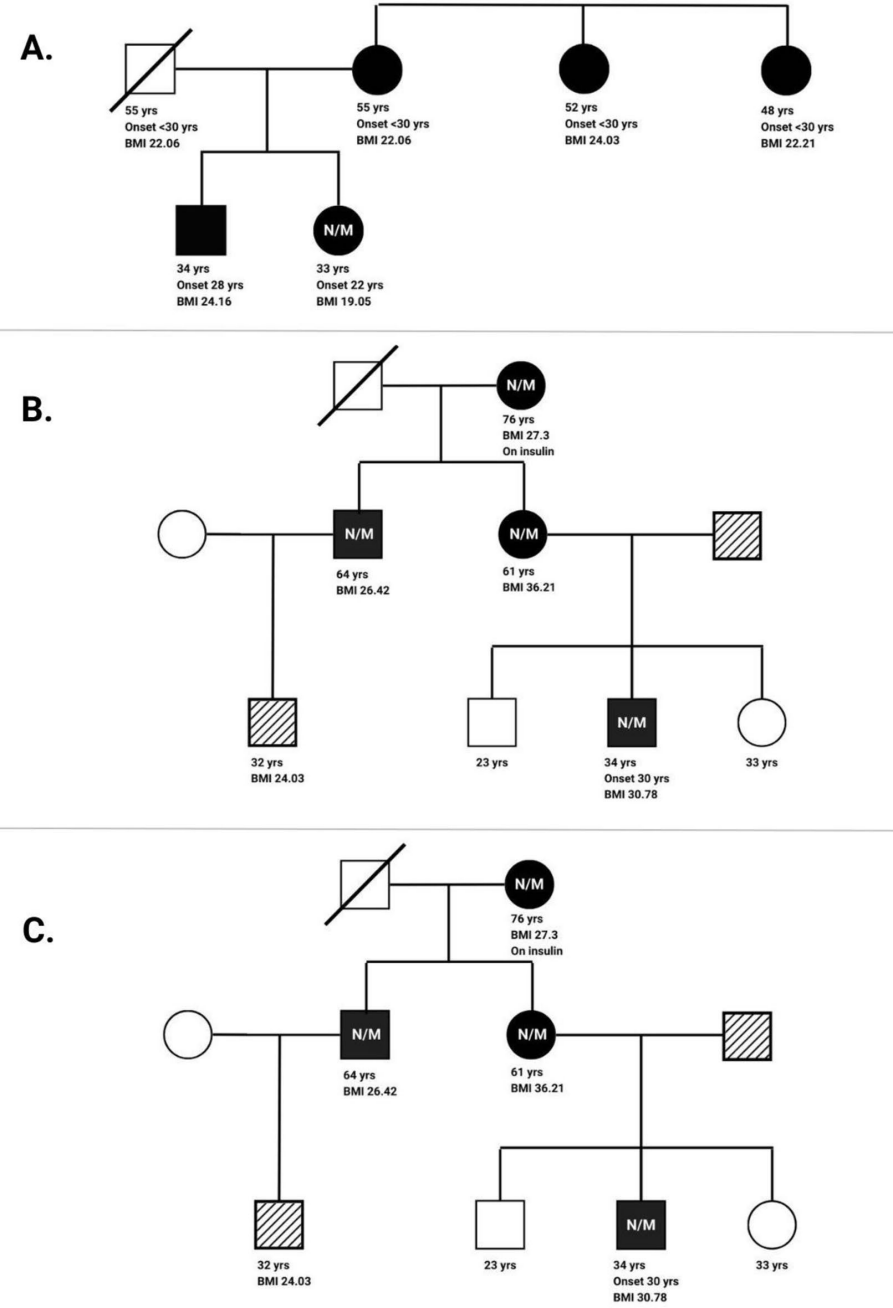
The family pedigree. Part (**A**), (**B**), and (**C**) related to HNF4A-MODY, HNF1A-MODY, HNF1B-MODY pedigree respectively. The square symbol represents male, circle represents female. The black symbols indicate diabetic individuals and symbols with oblique lines indicate pre-diabetics. N/M indicates a known carrier of the disease.

There was one *HNF4A* variant found, that had previously been classified as a pathogenic variant by ClinVar database. Additionally, this particular variant has been listed as an AD variant in Online Mendelian Inheritance in Man (OMIM) (125850). One proband was the carrier of this well-known missense variant (Val108Ile). A 33-year-old Iranian woman was diagnosed with diabetes at the age of 22 with a maternal family history of the disease. She had not been treated with any glucose-lowering agents and has a normal BMI of 19.05 kg/${\text{m}}^{2}$. Her older brother, who had a normal BMI, was also diagnosed with diabetes at the age of 28. Despite the lack of information regarding her father's glycemic status, her mother and two maternal aunts had young-onset diabetes and normal BMIs. Additionally, her mother had dyslipidemia (especially low HDL), hypertension, and diabetic retinopathy^3^. The traits of this pedigree are shown in Fig. 1, suggesting a chance of maternal transmission of the variation. Indeed, the potential AD transmission pattern and 100% disease penetrance indicate that it has a causative impact on MODY presentation in our population.

#### HNF1A-MODY

One *HNF1A* variant was found that had previously been reported as a pathogenic variant by ClinVar. Additionally, this particular variant was previously identified as a MODY-causing AD variation in OMIM (600496). One proband had this well-known frameshift variant (insertion p.Pro289fs). A 24-year-old Iranian woman with a strong diabetes familial history was diagnosed with diabetes at age 13. She wasn't receiving any glucose-lowering agents with poor glycemic control (FPG = 171 mg/dL). Her BMI was 22.73 kg/${\text{m}}^{2}$, which is considered normal. Despite her father being normoglycemic, her mother, her maternal grandmother, and three of her maternal uncles all developed diabetes before the age of 18 and required insulin treatment. All the maternal uncles were normal weight or slightly overweight. Furthermore, dyslipidemia was found in each of the aforementioned maternal family members. Additionally, the eldest uncle's history of angioplasty and the other uncle's history of diabetic retinopathy are indicative of the microvascular complications associated with *HNF1A-MODY*^3^. Figure 1 demonstrates the characteristics of the family pedigree. The potential AD pattern of transmission and complete disease penetrance (100%) of this variant shows that it has also a causative impact on MODY presentation in our population.

#### HNF1B-MODY

Between the two *HNF1B* variations, one was previously described as P/LP, and the other one was a potentially causal MODY variant with Uncertain Risk Allele in ClinVar classification. Regarding the previously described variant, the disease penetrance of this variant was 12.5% and none of the carriers showed the special clinical manifestations for Renal Cysts and Diabetes Syndrome (RCAD) related to *HNF1B-MODY*^3,30^. About the potentially causal MODY variant, four patients from a single family had this missense variation. A 76-year-old woman with diabetes who had been enrolled in the cohort trial was the first carrier. This variant was present in each of her two children, who were both diagnosed with diabetes. Her grandson, a 34-year-old male with diabetes who was first diagnosed at age 30, was the other carrier. The population's response to this variation revealed complete penetrance. Although AD transmission and complete disease penetrance were observed, as most carriers were overweight or obese with none of the symptoms of RCAD, more investigations are needed to confirm the causative impact of this variant. The family pedigree is available in Fig. 1.

Since the other MODY subtype carriers (*GCK-MODY, CEL-MODY, INS-MODY*) were not diabetic or didn’t show any AD inheritance for the phenotype in their families, it appears that these variants haven’t causative impact on MODY manifestation in our population.

### MODY genetic spectrum in Iran

Overall, there were 49 MODY variant carriers from 25 families in the TCGS cohort study. The mean age was 48.8 ± 17 years, 21 (42.8%) carriers were female. 12 (24.4%) were diabetic, 20 (40.8%) were pre-diabetic, 14 (28.5%) were non-diabetics and the glycemic status of 3 participants was unavailable. The mean age of onset for diabetic cases was 23.9 ± 5 years.

## Discussion

We identified seven MODY variants in 49 individuals from 25 families. Inclusively, six P/LP MODY variants were detected in 1.47% of diabetic participants. These variants were from six different genes including *HNF4A, GCK, HNF1A, HNF1B, CEL*, and *INS*. While variants in *INS-MODY* and *GCK-MODY* were more prevalent, variants in *HNF1A-MODY* and *HNF4A-MODY* exhibited complete disease penetrance. Moreover, one potential MODY variant located on *HNF1B* with an Uncertain Risk Allele was reported in 0.13% of diabetic participants. These findings warrant further discussion.

To enhance the prognosis of MODY, genetic testing is currently performed worldwide to facilitate predictions of the clinical course and prognosis of MODY^31^. The prevalence of MODY carriers varies among populations. The majority of studies conducted on European ancestry, and the scant research on Middle Eastern ancestries led to the conclusion that reported MODY variants cannot fully explain MODY cases in the Middle East^14,32,33^. This is while the Middle East and North Africa (MENA) region has the highest prevalence of diabetes globally^34^. Moreover, the significant load of genetic homozygosity due to the high rate of consanguineous marriages in the MENA region makes a remarkable distinctive genetic spectrum in this area^5,16^. Due to the unknown etiology and underreporting of MODY in the MENA region, lucrative academic and clinical research on MODY has been pursued in the Middle East^25,33^.

In Europe, MODY accounts for 1–5% of all diabetes mellitus cases^17^. We estimated the prevalence of P/LP variants of MODY at 1.47% of diabetes patients. Although in Europe *HNF4A-MODY*, *GCK-MODY*, and *HNF1A-MODY*, carry the most common pathogenic variants, accounting for > 80% of all monogenic diabetes*,* in the MENA region, MODY prevalence is mainly unexplored^26,35–38^. In our cohort, the prevalence of pathogenic variants on these genes were respectively, 0.006%, 0.080%, and 0.006%, which demonstrated a higher prevalence for *GCK-MODY*, and lower for *HNF1A-MODY* and *HNF4A-MODY* comparing to other cohorts, including UK biobank and Geisinger cohort. As TCGS is a 25-year-old family-based study representing all Iranian ethnicities, it is an unselected cohort, eliminating concerns about "clinical-referral ascertainment bias". Compared to other cohorts, derived from clinical settings or specific disease registries, the TCGS cohort's unselected nature provides a broader, more generalized understanding of penetrance in the general population^38^. According to our findings, *INS-MODY* was the most common type of MODY accounting for 46.6% of all variants in Iranian participants. However, *INS-MODY* variants were previously reported to be exceedingly rare in MODY patients worldwide^39,40^. Furthermore, *GCK-MODY* and *HNF1B-MODY* were the two next most common subtypes of MODY in the TCGS cohort study respectively. These differences can be attributed to the fact that although Iranians are closely related to nearby populations, they also exhibit unique genetic variation fall apart into a cluster of similar groups and several admixed ones, which is consistent with long-term genetic continuity, high levels of heterogeneity and consanguinity, and multiple historical language adoption events^41^. Furthermore, because the cohort is family-based and consanguineous marriage is common in the population, the transmission of variations across pedigrees could alter the population's genetic map. Taking into account the clinical criteria of MODY, 25 participants of TCGS met these clinical requirements. However, only 3 (12%) of them were detected with previously identified P/LP MODY variants (*HNF4A-MODY*, *HNF1A-MODY,* and *INS-MODY*). As evidenced by that 88% of participants meeting clinical criteria for MODY were devoid of known P/LP variants, it is plausible that novel MODY variants or variants which are benign in other ancestries could be pathologic in the Middle-Eastern population. This information underscores the significance of conducting population-based studies to identify prevalent MODY genes in non-European populations. In line with the reports of the second international consensus report on precision diabetes medicine 2023, which demonstrates that there is a major gap in the research for genetic detection of monogenic diabetes mellitus (MDM) in non-European origin populations^11^.

Concerning the clinical manifestations of different MODY subtypes, Although MODY carriers mostly showed normal BMI, we found a notable difference in BMI among MODY subtypes. In detail, the higher prevalence of obesity was discovered in *CEL-MODY* carriers. However, due to the rarity, the characteristic of this MODY subtype has not been completely elucidated^26^. Regarding *GCK-MODY*, these carriers typically exhibit mild hyperglycemia with an OGTT glucose increment of less than 60 mg/dl in European individuals. This was consistent with our finding on OGTT glucose increment and mean FPG (120 ± 65) of *GCK-MODY* carriers^42^. However, Chinese study reported a contrary finding in which 57% of participants experienced a high OGTT glucose increment^3,36^. On the other hand, *HNF1A-MODY* carriers mostly experience a large increment in blood glucose (> 80 mg/dl) after meals or during OGTT. This was compatible with our single *HNF1A-MODY* carrier laboratory results, which showed the glucose increment (FPG = 171mg/dl and OGTT = 260 mg/dl). About the clinical features of *HNF1B-MODY*, known as Renal cysts and diabetes syndrome (RCAD), this subtype is often accompanied by variable phenotypes including malformation of the pancreas, urogenital abnormalities, renal cysts, and neurocognitive defects. Although *HNF1B-MODY* carriers in our study didn’t report any neurocognitive and urogenital defects in their hospitalization records and cohort questionaries, diagnostic imaging may help diagnose co-occurring features of *HNF1B-MODY* in these variant carriers. Thus, Renal ultrasonography was offered to the patients and their relatives^43,44^. The detected variant for *HNF1A-MODY* in our study (chr12:120994313) was also reported previously. A recent study in the Kuwaiti population performed on 45 individuals with clinically diagnosed MODY, found this frameshift variant in one proband. After the diagnosis of *HNF1A-MODY*, the insulin therapy of the Kuwaiti carrier was replaced with Gliclazide, resulting in optimal glycemic control^5^. In our investigation, a single proband carried the identical variant. Although she was not receiving any blood glucose-lowering agents, her mother and all her maternal uncles were diabetic and under insulin treatment due to an early diagnosis of diabetes. This family was advised to visit an endocrinologist for possible treatment change and genetic consult for other family members.

One of the strengths of our study is that we have performed a gene-based cohort design to study the effect of rare variants on a particular phenotype. When studying the relationships between genotype and phenotype, it is important to examine the statistical occurrence of phenotypes in a group of known genotypes. Large population studies are required to investigate the disease penetrance, which aids in predicting the probability that a trait will be evident in carriers of the underlying alleles. Mentioned Kuwaiti study and other studies in Iran, firstly chose phenotypically possible MODY cases for detecting causal variants. This is while the reported variants may have a high allele frequency when evaluating the whole population genetic spectrum, which makes them unlikely to be causative for a monogenic rare disease^5,45–47^. Also, as some MODY subtypes like *GCK-MODY* mostly show mild hyperglycemia which is sometimes even undetectable, an exclusive clinical approach to select samples based on phenotype for finding causative variants seems inadequate. A founded variant can appear to be associated with the disease, regardless of whether the genotype has a functional effect on that health outcome in the whole population or not. Another strength of our study is that this is the first family-based cohort to evaluate MODY in the MENA region. Regarding similar works for the genetic of MODY in this region, a recent population-based study on 14,364 Qatari participants of the Qatar biobank, reported 22 previously-known MODY‐causing variants in 2.2% of subjects, and 28 potentially novel MODY‐causing variants in 1.14% subjects. Overall, discrepancies in reported variants among our MODY cases compared to those from Qatar could be attributed to variations in the study design. Despite the population-based design of both studies, as MODY typically shows an AD inheritance, a family-based design is necessary to better peruse familial transmission of the disease in family members^25^.

This study has some limitations as well. First, although the presented MODY phenotypic classification offers many positive aspects, there are still certain domains that could benefit from further clarification or improvement. The lack of C-peptide and autoantibody tests in this study constituted a limitation. The absence of concomitant islet autoimmunity or additional signs of insulin resistance and C-peptide levels may aid in ruling out T1DM even though MODY diagnosis is based on genetic testing. Second, although this is the first and sole cohort study on the genome of the Iranian population for monogenic diabetes, as the study is ongoing, whole genome sequencing of additional cases will be needed for better prevalence estimation and variant confirmation. Finally, additional research must be done to confirm the reported new variants' pathogenicity in other populations especially in the MENA region.

## Data Availability

Some part of the data used in this manuscript is available in the main text. Still, the whole data is available from the corresponding author on reasonable request.
